# The effectiveness of daily SMS reminders in pharmaceutical care of older adults on improving patients’ adherence to antihypertensive medication (SPPA): study protocol for a randomized controlled trial

**DOI:** 10.1186/s13063-017-2063-8

**Published:** 2017-07-18

**Authors:** Zuzana Haramiova, Michal Stasko, Martin Hulin, Tomas Tesar, Magdalena Kuzelova, Donald M. Morisky

**Affiliations:** 1 Department of Organization and Management of Pharmacy, Faculty of Pharmacy, Comenius University in Bratislava, Kalinciakova 8, 832 32 Bratislava, Slovak Republic; 2 Research Institute for Child Psychology and Pathopsychology, Cyprichova 42, 831 05 Bratislava, Slovak Republic; 3 Department of Pharmacology and Toxicology, Faculty of Pharmacy, Comenius University in Bratislava, Odbojarov 10, 832 32 Bratislava, Slovak Republic; 4 Department of Community Health Sciences, UCLA Fielding School of Public Health, 650 Charles E. Young Drive South, 46-071 CHS, Los Angeles, CA 90095-1772 USA

**Keywords:** mHealth, SMS reminders, Adherence, Antihypertensive drugs, Pharmacists, Cost-effectiveness

## Abstract

**Background:**

Despite a variety of efficient and cost-effective antihypertensive medication, hypertension remains a serious health and economic burden. High consumption of cardiovascular drugs in the Slovak Republic does result neither in better hypertension control nor in significant decrease in cardiovascular mortality. At the same time, Slovakia has alarmingly low patients’ adherence to medication intake. Studies have shown the efficiency of short messaging service (SMS) reminders to improve patients’ adherence and health outcomes at low costs. Since SMS is popular among Slovaks, this approach may be feasible also in Slovakia. The primary objective is to assess if daily SMS reminders of antihypertensive medication intake provided by pharmacists in addition to the standard pharmaceutical care increase the proportion of adherent older hypertensive ambulatory patients.

**Methods:**

The SPPA trial is a pragmatic randomized parallel group (1:1) trial in 300 older hypertensive patients carried out in community pharmacies in Slovakia. Trial pharmacies will be selected from all main regions of Slovakia. Trial intervention comprises daily personalized SMS reminders of medication intake embedded into usual pharmaceutical practice. The primary outcome is a combined adherence endpoint consisting of subjective self-reported medication adherence via the eight-item Morisky Medication Adherence Scale (MMAS-8) and objective pill count rate. Secondary outcomes include: change in the MMAS-8; comparison of adherence rates using pill count; change in systolic blood pressure; and patient satisfaction. Also, direct treatment costs will be evaluated and a cost-effectiveness analysis will be carried out.

**Discussion:**

The SPPA trial engages community pharmacists and mobile health (mHealth) technologies via evidence-based pharmaceutical care to efficiently and cost-effectively addresses current main healthcare challenges: high prevalence of hypertension; overconsumption of cardiovascular medicines; low adherence to medication treatment; and resulting uncontrolled blood pressure. The results may identify new possibilities and capacities in healthcare with low additional costs and high value to patients.

**Trial registration:**

ClinicalTrials.gov, NCT03105687. Registered on 07 March 2017.

**Electronic supplementary material:**

The online version of this article (doi:10.1186/s13063-017-2063-8) contains supplementary material, which is available to authorized users.

## Background

Cardiovascular diseases caused 17.7 million deaths worldwide [1], including 25,906 in Slovakia [2] in 2015, accounting for 31.3% and 48.13% of the overall mortality rate in the world and Slovakia, respectively. One of the main cardiovascular risk factors is hypertension [3]. Its prevalence reaches 30–45% among European countries [3, 4] as well as in Slovakia [5–7], increasing with age [8]. Health consequences of hypertension in older patients are more complex and severe [9]. Additionally, hypertension also represents a significant economic burden [10, 11]. In 2014, Slovakia had the third highest cardiovascular drugs utilization among OECD countries (683.4 DID; 37.6% of total drug consumption) [12]. A total of €155.9 million were spent on cardiovascular drugs [12], while complications resulting from insufficiently treated hypertension are even more expensive [13]. High consumption of cardiovascular drugs in Slovakia poses a significant economic burden both to the healthcare system and to the patients [14].

Despite a wide range of both efficient and cost-effective antihypertensive medicines, blood pressure control remains insufficient. Recent findings of the EURIKA study report only 50% blood pressure control among European countries [15]. National studies estimate that only 21–31% of Slovak patients have controlled hypertension [16, 17]. Poor adherence to antihypertensive medication intake belongs to the key risk factors of uncontrolled blood pressure [18, 19] resulting in increased risk of stroke, hospitalization, and premature death [20–22]. World Health Organization (WHO) estimates the overall adherence to medication intake in patients with chronic diseases at 50% [23, 24], while the latest European results show a varying adherence in the range of 26–70% [25]. Seriousness of this problem in Slovakia best demonstrate the results of local studies reporting 15–19% adherence rates (ARs) among Slovak patients [26–30]. Adherence to medication is a complex behavior influenced by multiple factors associated with the patient, healthcare providers, healthcare system, and specific treatment. These may result in intentional or unintentional patients’ non-adherence. While intentional non-adherence is a result of patients’ active decision not to take their medication as prescribed, unintentional non-adherence is caused by other factors such as forgetfulness, misunderstanding of the medication regimens, access to medication, or language barriers [31]. Several interventions to increase patients’ adherence to medication intake have been developed [32]. However, the majority of them are associated with high costs, time, and health professional resources, providing promising but uncertain and variable results [33]. Since the Slovak healthcare system lacks financial as well as professional human resources [34, 35], most of the adherence-increasing interventions are not feasible in Slovakia.

Technological progress has recently resulted in the implementation of various mobile technologies in healthcare to improve patients’ clinical outcomes as well as the effectiveness of healthcare system [36, 37]. Studies have confirmed the effectiveness of short message service (SMS) reminders on improving adherence to medication intake, health status, and attendance at medical appointments of patients with infectious diseases [38–48]. Latest systematic reviews suggest that SMS reminders may also be effective in the management of chronic diseases [49, 50]. In 2015, Global System for Mobile communication network covered 92.0% of the Slovak territory and 100.0% of the Slovak population [51]. Additionally, national telecommunication statistics report 491.6 SMS sent per one inhabitant in 2015, demonstrating that SMS are a popular means of communication among Slovaks [52]. Considering all previously mentioned facts, we hypothesize that SMS reminders present a suitable approach to improve patients’ adherence to medication intake in Slovakia.

Pharmacists play a vital role in the healthcare system and can substantially improve the value of pharmacotherapy [53–55]. Studies have shown that pharmacists’ interventions in chronic cardiovascular disease management significantly improve patients’ outcomes [56–58]. Furthermore, local studies have proven that high-quality pharmaceutical care improves both the adherence to medication intake and blood pressure control in hypertensive patients [26, 59, 60].

The need of a multidisciplinary approach towards poor adherence to medication intake is being widely stressed and encouraged [23, 61, 62]. At the same time, the WHO concludes that improving patients’ adherence may have a greater effect on the health than any other improvement in therapy [23]. Therefore, the presented pragmatic trial explores the possibilities of mobile health (mHealth) interventions provided by pharmacists in improving patients’ adherence to antihypertensive medication intake in Slovakia. We hypothesize that personalized daily SMS reminders of antihypertensive medication intake provided by pharmacists in addition to standard pharmaceutical care (sPhC) will increase the proportion of adherent older ambulatory patients with hypertension from 30% to 49%.

## Methods

### Trial objectives

The primary objective of the SPPA trial is to assess if daily SMS reminders of antihypertensive medication intake provided by pharmacists in addition to the sPhC increase the proportion of adherent older hypertensive ambulatory patients.

Secondary objectives include: (1) comparing the change of self-reported adherence using the eight-item Morisky Medication Adherence Scale (MMAS-8); (2) comparing ARs measured via pill count between trial groups; (3) comparing the change in systolic blood pressure; (4) investigating the effect of SMS reminders on improving patients’ adherence and systolic blood pressure control (treatment effects) across subgroups; and (5) collecting and reporting patients’ satisfaction with daily SMS reminders.

We will report up-to-date ARs of older hypertensive patients in Slovakia and assess the impact of sociodemographic characteristics on patients’ adherence to antihypertensive medicines. Furthermore, we will evaluate the MMAS-8 questionnaire in our trial population. Additionally, we will carry out a simple cost-effectiveness analysis from the payers’ perspective.

### Trial design

The SPPA trial is designed as a multicenter assessor-blinded, controlled, randomized, superiority, pragmatic trial to assess the effectiveness of daily SMS reminders of antihypertensive medication provided by pharmacists in addition to sPhC compared with sPhC only. Patients will be individually randomized into two parallel groups in a 1:1 allocation ratio. Participants in the intervention group will receive daily SMS reminders of medication intake, whereas participants in the control group will receive sPhC only. The trial will last three months with a recruitment period of two months. The Standard Protocol Items: Recommendations for Interventional Trials (SPIRIT) checklist for study protocols [63] is provided in Additional file 1. Details about the pragmatic design of the SPPA trial are provided in the PRagmatic Explanatory Continuum Indicator Summary (PRECIS-2) table of scores for trial domains and the PRECIS-2 wheel scheme [64] in Additional file 2.

### Trial setting

The SPPA trial will be conducted in six or more community pharmacies in the Slovak Republic located in all main Slovak regions. Furthermore, half of the pharmacies will be selected from rural and half from urban regions. This selection process will ensure better generalizability and high external validity of trial results for the Slovak population of older hypertensive patients.

### Study population

Trial population consists of hypertensive ambulatory patients aged 55 years or older who are taking antihypertensive medication for at least one year without any discontinuation. Based on the trials’ pragmatic design, eligibility criteria are liberal and can be assessed solely using the information available to the pharmacists.

### Inclusion criteria

1. Age ≥ 55 years (from the day of the 55th birthday inclusive);
2. Diagnosis of primary hypertension (I10 according to ICD-10 [65]);
3. Filling of antihypertensive prescription(s) at trial recruitment (Visit 1);
4. Duration of antihypertensive drug treatment for at least one year without any discontinuation;
5. Ownership of a mobile phone for personal use with the ability to open and read SMS;
6. Understanding of Slovak language on native-speaker level;
7. Informed consent for participation in the clinical trial and personally signed informed consent form.

### Exclusion criteria

Exclusion criteria assessed prior to patient enrolment:

1. Planned hospitalization during the trial period;
2. Biological impairment affecting the ability to read the SMS (e.g. loss of vision, visual field cuts, aphasia);
3. Living in the same household with another trial participant;
4. Participation in another clinical trial.

Exclusion criteria assessed after patient enrolment:

1. Hospitalization during the trial period;
2. Patient informs he/she will not be able to participate in the trial;
3. Withdrawal of informed consent.

We have strived to maintain broad eligibility criteria to ensure our trial population best reflects the real-world population of patients for whom the intervention is intended. Nevertheless, we accepted some inclusion and exclusion criteria, which can strengthen the validity of the trial’s results while not introducing selection bias. We focus on patients with primary hypertension to limit the confounding influence of secondary hypertension causes both on patients’ adherence to medication and resulting blood pressure control. Patients with other co-morbidities are eligible to participate in the study. We will recruit patients while filing their prescription at the trial pharmacy. According to Slovak health legislation, physicians are allowed to prescribe medication to chronic patients for a maximum of three months. Hence, patients who are collecting their medicines at the day of the recruitment will in most cases begin to take the pills out of the collected medication package on the next day (first day of the intervention), enabling the precise calculation of pill count rates. Furthermore, chronic patients in Slovakia visit the pharmacy usually in three-month cycles. We will include only patients who take their antihypertensive medication for at least one year because these patients are considered to have reached their acceptance phase of medicinal treatment and consequently are at lower risk of early therapy discontinuation due to non-acceptance [66]. Trial participants hospitalized during the trial will be excluded since during their hospital stay healthcare providers will manage their medication resulting in the redundancy of SMS reminders.

### Trial groups

#### Control group

Participants in the control group will receive sPhC according to the principles of Good Pharmaceutical Practice and national Slovak legislation requirements [53, 54, 67, 68] only. Trial pharmacists will provide the sPhC to the patients while dispensing their medicines at the trial pharmacy. Participants in the control group will also receive a welcome SMS one day after enrolment and an end-of-trial SMS three months after the enrolment (Table 1).

**Table 1:** SMS messages in the SPPA clinical trial

SMS	Time of receipt	Content of the SMS
Welcome SMS	Day after enrolment	“Welcome in the SPPA trial. We would like to thank you for participating in this clinical trial. Your participation might help to improve health of other patients as well as the Slovak health care system. Your SPPA trial research team.”
Sample SMS reminder (intervention group only)	Daily for 3 months; starting the day after enrollment	“Please, be reminded to take your blood pressure-lowering drug(s): <DRUG NAME & STRENGHT>: <DOSAGE>, <FREQUENCY>, <FURTHER INSTRUCTIONS, IF APPLICABLE>. Thank you very much for taking care of your health. Your SPPA trial research team.”
End-of-trial SMS	3 months after the enrolment	“Thank you very much for your participation in the SPPA trial. Your prescheduled appointment at the trial pharmacy is on < DATE>, please bring your marked drug package(s) with you. We will be looking forward to your visit. Your SPPA trial research team.”

#### Intervention group

Participants in the intervention group will receive sPhC provided by the trial pharmacist, the welcome SMS, and the end-of-trial SMS. Additionally, they will be receiving the investigated intervention, daily SMS reminders of antihypertensive medication intake, for three months. Behavioral perspective of the content and the framing of SMS reminders are not yet sufficiently understood [69]; therefore, we will focus on simple information provision according to the national and international pharmaceutical regulations and guidelines [53, 54, 67, 68]. The content of the SMS reminders will be personalized for each participant in terms of drug name, strength, dosage, frequency, and other applicable specifications. Complying with the pragmatic design of the trial, the SMS reminder structure follows information provided during the usual drug dispensation and counseling process as laid down by the Slovak national decree No. 129/2012 [67]. Thus, most of the information are available on the prescription and all of the collected data are already a well-established and required part of the sPhC in Slovakia. Furthermore, simple structure of the SMS reminder allows for future reproducibility. Table 1 shows the content of sample SMS reminders.

Delivery times will be customized to patients’ individual preferences. We have chosen the length of the intervention to be three months for two main reasons. First, previous studies have confirmed that a three-month period is sufficient to detect a significant change in medication adherence [70–72]. Second, according to Slovak law [68], chronic patients can receive their medication for a maximum of three months. Afterwards, they attend a regular medical visit, receive a new prescription, and thus visit the pharmacy usually in three months cycles. We believe this will increase the retention rate. For dissemination and coordination of the SMS reminders we will use FrontlineSMS, open access software, and a local operator Subscriber Identification Module Card. An external unblinded trial pharmacist will create, schedule, and send the SMS reminders according to the case report forms. The external unblinded trial pharmacist will have direct contact with neither the trial participants nor the trial pharmacists.

### Patient recruitment

Participants will be recruited in trial pharmacies while filling their prescriptions. Trial pharmacists will screen patients according to their prescription information (age, diagnosis, and medication). If deemed eligible, they will inform the patients about the SPPA trial and their potential participation.

Trial pharmacies will be selected according to their recruitment potential (number of hypertensive patients visiting the pharmacy per month). In case a center will not be able to reach the predefined enrolment target, an additional trial center, comparable in region characteristics, will be trained and initiated to complete the enrolment.

### Randomization, allocation, and concealment

Participants will be randomly assigned to either control or intervention group with a 1:1 allocation ratio. The external unblinded trial pharmacist will generate a simple randomization and allocation list with randomization numbers (patient trial codes) stratified by gender for each trial center. Freely available online software, Research Randomizer, will be used for this purpose [73]. The external unblinded trial pharmacist will provide each trial pharmacy with equal number of sequentially numbered, opaque, sealed randomization envelopes for male (blue) and female participants (red). Trial pharmacists will recruit patients in the natural, unpredictable order as they enter the pharmacy and agree to participate in the trial. After signing the informed consent form, trial pharmacists will assign a patient trial code to each participant from a randomization envelope selected in ascending order.

### Blinding

Due to the nature of the trial, it is not possible for the patients to be blinded. Trial pharmacists will inform the participants about the purpose of the trial and their random allocation to either receiving daily SMS reminders or not and will ask them not to disclose their allocation to the trial pharmacist or research team until the end of their follow-up visit. Trial pharmacists, performing blood pressure measurements, pill count calculations, and completing the MMAS-8 questionnaires with the participants (the assessors), will be blinded to the allocation of the intervention. The statistician will receive the final anonymized data after the end of trial and database lock. The project leader is the main contact person for trial participants in case of potential problems. Thus, it is possible that during these contacts patient allocation may be disclosed. However, since the project leader is involved neither in the assessment of the trial outcomes nor in the final statistical analysis, this potential partial unblinding will have no impact on the trial results. Other research members are blinded to the intervention allocation.

### Data collection, handling, and monitoring

Trial data will be collected at Visit 1 (day of patient enrolment) and at Visit 2 (follow-up visit after three months). Table 2 provides and overview of all case report forms that will be used in the trial for data collection. Furthermore, we will actively seek and collect signals of adverse events associated with antihypertensive medication intake reported by the participants. We will also collect anonymous reasons for participation refusal and discontinuation of participation. All trial forms and materials constituting the Appendixes of the SPPA trial protocol are available in Additional file 3.

**Table 2:** Overview of Case Report Forms in the SPPA trial

Case Report Form/Questionnaire	Details	Time Point
Case Report Form Visit 1 – PART A	Antihypertensive medication specification Mobile phone number for SMS reminders Preferred time of SMS reminder	Visit 1
Case Report Form Visit 1 – PART B	Blood pressure measurement	Visit 1
Case Report Form Visit 1 – PART C	Medication adherence measurement via MMAS-8	Visit 1
Case Report Form Visit 1 – PART D	Patient information (Sociodemographic and health information, information on mobile phone usage)	Visit 1
Case Report Form Visit 2 – PART A	Medication adherence measurement via pill count	Visit 2
Case Report Form Visit 2 – PART B	Blood pressure measurement	Visit 2
Case Report Form Visit 2 – PART C	Medication adherence measurement via MMAS-8	Visit 2

**Fig. 1:**
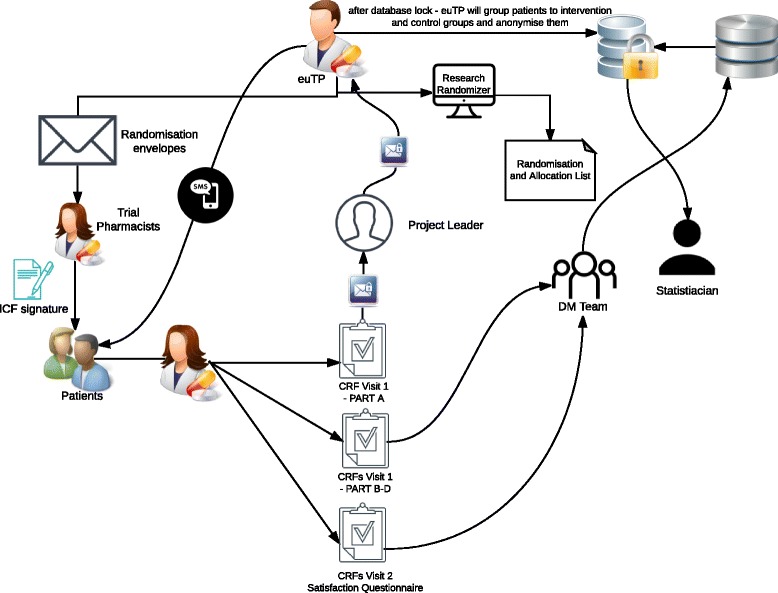
Trial data procedures and data workflow diagram. *euTP* external unblinded trial pharmacist, *ICF* informed consent form, *CRF* case report form, *DM* data management

During the recruitment period, the trial pharmacist will send password-protected scans of the Case Report Form Visit 1 – PART A and the Screening and Enrollment Log to the project leader via e-mail. The project leader will then forward the antihypertensive medication specification, mobile phone number, and preferred time for SMS reminder in a password-protected document to the external unblinded trial pharmacist via e-mail. Trial pharmacists will send copies of all trial forms, except Case Report Form Visit 1 – PART A, in sealed envelopes via recorded delivery to the research office located at Faculty of Pharmacy, Comenius University in Bratislava on a monthly basis. A separate data management team, whose members are not part of the research team, will enter the data into the pre-prepared, password-protected Microsoft Access Database. Two members of the data management team will do the data entry on a double-entry basis independently to ensure excellent quality of the data. Figure 1 depicts all trial data procedures and data workflow.

### Outcome measures

#### Primary outcome measure

Our primary outcome is the proportion of adherent patients in the intervention group after three months of receiving SMS reminders of medication intake. For these purposes, we will assess patients’ adherence via combined adherence endpoint consisting of self-reported medication adherence measured via MMAS-8 and pill count rate (%).

The eight-item MMAS questionnaire, on which the MMAS-8 is based, evaluated hypertensive patients. It showed good reliability (α = 0.83) as well as sensitivity (93%) and specificity (53%) in the identification of lower versus higher adherence to antihypertensive medication. Additionally, the association between MMAS-8 and blood pressure control was proved statistically significant (*p* < 0.05) [19]. Each question aims at assessing a specific medication-taking behavior, either intentional or unintentional. The first seven questions have dichotomous answers (yes/no), whereas the last question offers a five-point Likert response. The range of the scores is from 0 to 8 with 0 being low adherence and 8 indicating full medication adherence [19, 74].

Pill count is a simple objective indirect measurement of patients’ adherence to medication intake [75]. AR will be calculated using the following equation:

$$ \mathrm{A}\mathrm{R}\left(\%\right)=\left(\frac{\mathrm{No}.\mathrm{of}\;\mathrm{pills}\;\mathrm{actually}\;\mathrm{taken}}{\mathrm{No}.\mathrm{of}\;\mathrm{pills}\;\mathrm{that}\;\mathrm{should}\;\mathrm{have}\;\mathrm{been}\;\mathrm{taken}}\right)\times 100 $$

No specific guidelines exist on how to assess medication adherence [76]; therefore, we based our categorization of a patient as being “adherent” or “non-adherent” on the previous literature and expert opinions. Our study team agreed that a patient at the end of the trial will be considered as either “adherent,” having MMAS-8 ≥ 6 [19] and pill count rate ≥ 80% [77], or “non-adherent” if one or both of the previous conditions are not met. Table 3 contains details on our primary outcome measure.

**Table 3:** Primary outcome Combined adherence endpoint

Level	
Domain	Combined medication adherence
Specific measurement variable	Adherence status (dichotomous) adherent: MMAS-8 score ≥6 and pill count rate ≥80% non-adherent: MMAS-8 score <6 and/or pill count rate <80%
Analysis Metric	Final value (Visit 2)
Method of aggregation	Proportion of adherent patients (%)
Time point of measurement	At Visit 2 (follow-up visit after 3 months of intervention period)

#### Secondary outcome measures

Table 4 summarizes the details of our secondary outcome measures. We will also evaluate the clinical outcome – blood pressure, which is highly emphasized to provide information on the relationship between medication adherence and blood pressure control [33]. Considering our trial population, consisting of older patients, we have decided to evaluate only systolic blood pressure, not diastolic blood pressure, in our secondary clinical outcome. First, diastolic blood pressure is known to paradoxically decrease with increasing age in patients aged above 55 years [78]. Second, diastolic blood pressure has shown to be less predictive in regard to decrease in cardiovascular morbidity and mortality [79]. We prepared a detailed guidance for blood pressure measurement (SPPA Scheme for Blood Pressure Measurement is available within Additional file 3) based on the current international recommendations for blood pressure measurement [4, 80]. Trial pharmacists will be re-trained on all steps and will comply with the SPPA Scheme for Blood Pressure Measurement during the whole trial, which will ensure that all blood pressure measurements will be performed uniformly.

**Table 4:** Secondary outcomes

Secondary outcome	Change in medians of MMAS-8	Mean adherence rate after 3 months	Mean change in systolic blood pressure
Domain	Self-reported medication adherence (MMAS-8 questionnaire)	Medication adherence (pill count)	Blood pressure (manometer)
Specific measurement variable	MMAS-8 (categorical, ordinal)	Adherence rate in % (numerical, continuous)	Systolic blood pressure in mmHg (numerical, continuous)
Analysis Metric	Change from baseline (Visit 1)	Final value (Visit 2)	Change from baseline (Visit 1)
Method of aggregation	Median	Mean	Mean
Time point of measurement	Visit 1 Visit 2	Visit 2	Visit 1 Visit 2

We will also collect information on patients’ satisfaction with the intervention using our brief satisfaction questionnaire. The satisfaction questionnaire is based on previous studies [43, 71, 81, 82], stressing out the most important aspects for tailoring future mHealth trials to best reflect patients’ needs. Four pharmacists and a social psychologist assessed face validity of the questionnaire. Consequently, five patients aged above 55 years assessed the questionnaire for clarity, simplicity, and understanding. We adjusted the questionnaire according to their recommendations and comments. The final Satisfaction Questionnaire is included within Additional file 3.

#### Other outcome measures

We will collect the direct treatment costs associated with antihypertensive medication for each participant according to the current list of categorized drugs issued by the Ministry of Health of the Slovak Republic [83] on a monthly basis. We will actively collect reported signals of adverse events associated with antihypertensive medication during the whole trial duration.

### Participant timeline

**Fig. 2:**
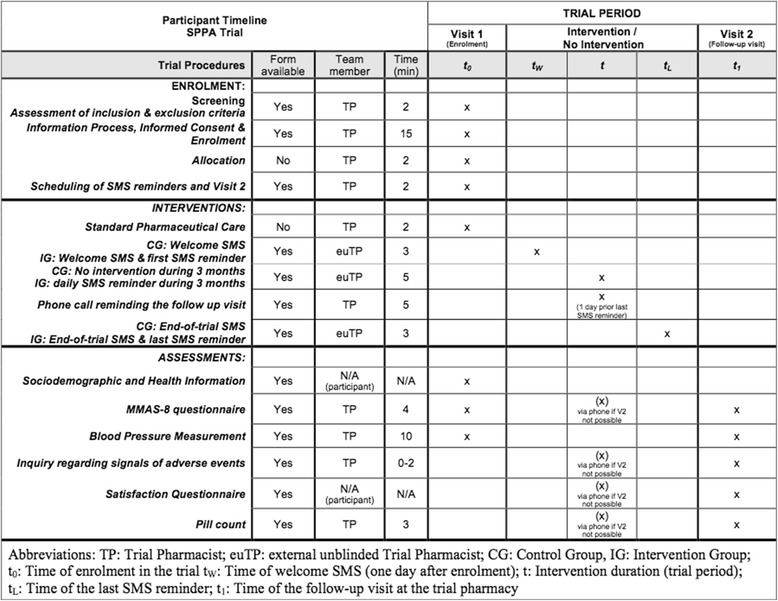
SPIRIT figure. *TP* trial pharmacist, *euTP* external unblinded trial pharmacist, *CG* control group, *IG* intervention group, *t*_*0*_ time of enrolment in the trial, *t*_*W*_ time of welcome SMS (one day after enrolment), *t* intervention duration (trial period), *t*_*L*_ time of the last SMS reminder, *t*_*1*_ time of the follow-up visit at the trial pharmacy

Figure 2 summarizes all trial procedures.

To minimize attrition of participants, trial pharmacists will contact all participants via phone call one day prior to their pre-scheduled follow-up visit as a reminder. During this phone call, the follow-up visit may be rescheduled to suit patient’s needs. If participants are not able to attend the follow-up visit at all, they will be asked to perform the pill count, MMAS-8, and the satisfaction questionnaire via phone call.

### Pilot test

Prior to trial initiation, a pilot test will be performed in one of the trial pharmacies with 20 patients, all receiving the intervention for two weeks. These patients will not be included in the final trial analysis. All procedures will be performed according to the SPPA trial protocol. The trial’s eligibility criteria and participation refusal rate will be assessed to ensure attainability of the planned sample size. SMS software will be tested, monitoring for any systematic errors, and a risk management protocol will be prepared. All data collection forms will be assessed with regard to comprehensiveness for trial participants and practical use for trial pharmacists, respectively. In cooperation with trial pharmacists and participants, we will evaluate the SMS wording for comprehensiveness, practicality, and patients’ perceptions. The data management team will test the data management pathway. Detected issues will be processed and the trial protocol will be revised, if necessary.

### Sample size calculation

In the recent multinational survey, adherence prevalence in European countries was in the range of 30–76% [25]. Given the results of the latest national study reporting even lower ARs [28], we assume 30% adherence prevalence in the control group. According to a meta-analysis of randomized clinical trials, SMS reminders had the potential to double the odds ratio (OR) of medication adherence (OR = 2.11; 95% confidence interval [CI] = 1.52–2.93; *p* < 0.001) in cardiovascular patients. The weighted mean effect size was d = 0.41 (95% CI = 0.23–0.59). After adjusting for publication bias, the odds of being adherent in the group receiving SMS reminders was 68% higher than in the control group (OR = 1.68; 95% CI = 1.18–2.39) [50]. Results of the StAR trial carried out in a hypertensive population support these results, reporting an OR of 1.86 (95% CI = 1.20–2.16; *p* = 0.002) for improved adherence in patients receiving SMS reminders [84]. Therefore, we hypothesize that SMS reminders of antihypertensive medication intake can improve the proportion of adherent older patients in Slovakia from 30% to 49% given an effect size of w = 0.2 (equivalent to d = 0.4). Using a χ2 test of independence, a total of 264 patients are needed to reject the null hypothesis that the adherence proportions in both groups are equal with a power of 0.9 and alpha of 0.05. Considering approximately a 10% attrition rate, we plan to enroll 300 patients (150 in each group). Previous studies reported attrition rates of 3.2% [71], 12.8% [84], and 19.0% [70]; while the major reason for loss of participants was unwillingness or impossibility to attend the follow-up visit(s). Slovak chronic hypertensive patients are already used to regular visits of pharmacies in approximately three-month cycles. Since our trial is embedded within usual pharmaceutical care provision, we expect a lower drop-out, as in the EmPhAsIS study [85].

The planned sample size is sufficient to detect a difference in adherence of 1 point on the MMAS-8 scale, which is our secondary outcome. Provided that the median MMAS-8 score in the control group is 4 and in the intervention group 5 (SD = 1.8) [30], using a two-tailed t-test, we need 172 participants to achieve a power of 95% and significance level of 5%. For our secondary clinical outcome (mean change in systolic blood pressure), this sample size lacks sufficient power to detect a clinically meaningful change of at least 5 mmHg (SD = 20) with a power = 0.9, alpha = 0.05 (using either mixed analysis of variance or analysis of covariance test). A larger sample is currently not feasible; however, the SPPA trial focuses primarily on adherence to medication. If the results of this trial show sufficient effectiveness of SMS reminders in increasing prevalence of adherent older hypertensive patients and patients’ satisfaction is high, we will conduct a larger study focusing on clinical outcomes. Sample size calculations were carried out using G*Power software version 3.1.9.2.

### Statistical analysis

We will conduct all analyses according to the intention-to-treat principle, striving to collect data from all participants. In case of missing data, data imputation techniques (multivariate imputation via chained equations) will be used. Predictive mean matching and logistic regression [86] will be used for continuous (e.g. systolic blood pressure) and categorical (e.g. MMAS-8) variables, respectively. For comparison, a per-protocol analysis will also be carried out. We will comprehensively report all missing data along with the reasons why these data are missing and explore their patterns. This information will help us to appropriately adjust future study protocols. No interim analysis is planned. Descriptive statistics will be reported as means (SD) or medians (interquartile range) for continuous variables and proportions for categorical variables. All statistical analyses will be carried out with a 5% type I error rate.

Results of the SPPA trial will be reported following the Consolidated Standards of Reporting Trials (CONSORT) 2010 statement [87, 88] and the Guidelines for reporting of health interventions using mobile phones: mobile health (mHealth) evidence reporting and assessment (mERA) checklist [36]. All analyses will be conducted with R software [89] using the basic statistical functions as well as robust functions from the package WRS2 [90] and for plotting and graphs the ggplot2 package [91].

#### Primary outcome analysis

In the primary analysis, we will compare the proportion of adherent patients according to the combined adherence endpoint after three months in the control and intervention groups using the Chi-square test. ORs and their effect sizes together with the corresponding 95% CIs will be calculated. The absolute effect size will be reported as the number needed to treat adjusted for the purposes of this trial to “number needed to remind.” To accommodate the pre/post design for the primary binary outcome, two samples McNemar test will be used [92].

#### Secondary outcome measures

##### Change in medians of MMAS-8 after three months

The difference in median MMAS-8 scores reported after three months between the intervention and the control group will be calculated. The effect size and the corresponding 95% CI will be reported. We will use a specific independent sample t-test on robust location measure (median) implemented in the function “medpb2” in Randy Wilcox’s R package WRS2 [93].

#### Mean adherence rates after three months calculated via pill count

The mean difference in adherence reported as AR (%) measured at Visit 2 between the intervention and the control groups will be calculated. The effect size and the corresponding 95% CI will be reported. We will use a two-tailed t-test to compare the difference between these independent means as well as their robust versions that are recommended when conditions for these tests are violated while preserving high statistical power.

#### Mean change in systolic blood pressure

For the clinical secondary outcome, the results of a mixed analysis of variance (suitable to assess change in pre and post measurements) and analysis of covariance (suitable to assess the differences in pre and post measurements) as well as their robust versions, that are recommended when conditions for these tests are violated while preserving high statistical power, will be compared [94].

#### Patients’ satisfaction with SMS reminders

First, we will analyze the psychometric quality of the questionnaire items. Cronbach’s alpha, McDonalds’s hierarchical omega, as well as Mokken scale analysis [95] will be used to find out if all items have sufficient quality to be included in the final scale. In case of significant deficiencies, item(s) will be excluded. Second, using the final scale, we will analyze the impact of patient satisfaction level on the change of adherence and systolic blood pressure values via multivariate linear regression, controlling for impact of sex, age, education, etc. This way we strive to find out if the overall satisfaction level of patients is a significant predictor variable for the change in ARs and/or systolic blood pressure values.

#### Subgroup analysis

We will conduct a subgroup analysis of the SMS reminders’ effect on improving adherence and systolic blood pressure (treatment effects) across subgroups based on: sex, baseline systolic blood pressure, number of years with hypertension, education level, number of daily antihypertensive medications, frequency of antihypertensive medication intake, and baseline self-reported AR according to MMAS-8. We will use the QUINT (Qualitative INteraction Trees) package in R software for a qualitative subgroup analysis to detect different direction of impact for various subgroups [96].

#### Other outcomes analyses

We will assess internal consistency of the MMAS-8 scale in our trial population by Cronbach’s alpha coefficient and reliability of the summed score by McDonald’s hierarchical omega, which has been shown to be a superior measure to Cronbach’s alpha [97]. We will also provide the measures of sensitivity and specificity of MMAS-8 related to correct prediction of high blood pressure. Of interest is also the relationship between two measures of adherence (MMAS-8 and pill count), which will be analyzed using correlation coefficients (Pearson and Kendall tau). Additionally, regression techniques to analyze the impact of co-variables, such as: age, gender, education level, social status, place of residence, number of daily doses, co-medication, co-morbidities (concomitant diseases, smoking status), and pharmacy on patients’ adherence will be applied.

Since we are interested in providing relevant information for the decision-making process, we will conduct a simple cost-effectiveness analysis. Here, we will focus only on the differential costs that depend on the choice of alternative care (usual pharmaceutical care or SMS reminder).

Finally, we will collect, report, and perform a qualitative analysis of the refusal to participation in the trial and withdrawal from the trial rates.

## Discussion

The SPPA trial protocol was designed to answer the research question whether personalized daily SMS reminders of antihypertensive medication intake, provided by pharmacists in addition to sPhC, increase the proportion of adherent older hypertensive ambulatory patients.

We aimed to develop a pragmatic trial that is not only able to prove the effectiveness of SMS reminders in improving patients’ adherence to medication intake in specific trial conditions, but can be easily implemented in daily practice after end of the trial. For this purpose, we have extensively used the innovative PRECIS-2 tool [64] thorough the whole trial design period. SPPA trial scored high in most of the trial domains (Additional file 2).

Studies have shown the potential efficacy of SMS reminders in improving adherence to medications as well as health outcomes in patients with infections and chronic diseases. Now, it is necessary to investigate the effect of SMS reminders in specific communities and populations [69, 84]. Our trial addresses these needs focusing on older patients within a community pharmacy setting. Prevalence of hypertension as well as the overall cardiovascular risk increases sharply with age while drug regimens become more complex. Thus, older patients are at higher risk of unintentional adherence, which is the leading cause of non-adherence among Slovak patients [98]. Consequently, older hypertensive patients are at higher risk for uncontrolled blood pressure due to non-adherence to medication treatment and could significantly benefit from this mHealth service.

We have decided to deliver the intervention, SMS reminders of medication intake, via publicly accessible online software that is easy to operate, its use is not limited for the current trial, and it is financially affordable also in low-resource settings.

Even though in the current trial SMS reminders will be sent out centrally by the external unblinded trial pharmacist, the operation of the system is intuitive and does not require extensive training of the pharmacists. We have agreed on central SMS sending to enable blinding of the trial pharmacists (assessors) to patient allocation.

Recent development in mHeath technologies has shown their effectiveness in addressing complex health issues. However, Slovakia, like many other countries, is characterized by shortage of healthcare professionals. Therefore, physicians or nurses have limited time capacities for implementation of mHealth technologies in healthcare. Simultaneously, pharmacists are highly trained healthcare professionals that are currently redefining their professional position in healthcare, mostly due to the industrialization of pharmacy. As a result, the importance of patient-centered pharmaceutical care [53] is dynamically growing. Pharmacists are considered to be experts on pharmaceuticals and patient consultation on medicinal therapy management. Also, pharmacists are already obliged by the international guidelines [54] as well as the Slovak legislation [67, 68] to provide patients with all necessary information on medication to ensure their safe and efficacious treatment. Additionally, pharmacists are the last healthcare professionals that patients consult in the drug chain process. Thus, we believe that pharmacists have the ideal predisposition and capacities to provide certain mHealth services, such as SMS reminders of medication intake, as a part of their daily practice. In order to strengthen the pragmatic design and implementation into daily practice, we designed the SMS reminders in the SPPA trial to mirror the structure of the dispensation information currently provided to the patients according to the Slovak regulations [67]. Consequently, the SMS reminders are aimed at improving non-adherence due to forgetfulness (unintentional), stress essential information regarding medicines, and support habit formation. We also believe that SMS reminders will strengthen the patient–pharmacist relationship, which is crucial for effective healthcare provision.

Our primary objective is to assess patients’ adherence according to the combined adherence endpoint consisting of self-reported medication adherence measured via MMAS-8 and pill count. Some researchers object that self-reported adherence questionnaires, such as the MMAS-8 used in this trial, tend to be subjectively biased [99, 100]. On the other hand, they use a highly patient-oriented approach that enables provision of feedback regarding adherence behavior and point out reasons for poor medication adherence [19]. Studies have shown that results of self-reported adherence questionnaires are correlated with the results of medication event monitoring systems. Even though medication event monitoring systems enable detailed monitoring of medication adherence, they are financially unsustainable in everyday practice and often provide also erroneous measures [101]. Pill count is an objective adherence measurement, although, if used alone it also may be biased [75]. Therefore, in the SPPA trial we combine two separate, simple adherence measurements suitable for the pragmatic design of the trial. We believe that combining two independent adherence measurements will provide a more reliable and valid outcome.

We will collect patient reported outcomes in regard to their perception of and satisfaction with the SMS reminders using a questionnaire designed by our research team. We will also analyze the psychometric properties of this satisfaction questionnaire for use in future studies.

SPPA trial will provide currently lacking up-to-date data on the adherence of older hypertensive patients in Slovakia, including evaluation of the internal consistency and reliability of the MMAS-8 questionnaire in our trial population. Additionally, we will collect and report anonymous reasons for refusal of participation in the trial as well as reasons for withdrawal from trial to guide future clinical trials to best address the needs of the patient community.

We put very high accent on documentation quality and data transparency. Accordingly, we will document all contacts between patients and research team as well as all problems and issues in form of contact reports. With our trial, we want to contribute to public research data sharing towards open science. Therefore, all anonymized data forms and contact reports will be made publicly available on the Open Science Framework platform [102].

The main limitation of the SPPA trial is that our current sample size lacks statistical power to detect significant change in clinical outcome, the systolic blood pressure. Also, the SPPA trial is not designed to focus on other important clinical outcomes such as cardiovascular risk reduction, prevention of cardiovascular events, or overall cardiovascular mortality. Furthermore, we formulate the SMS content solely to remind patients of their medication intake, not to educate or motivate them in any specific way, as some previous studies have intended [70, 84, 103]. Thus, our investigated intervention addresses only unintentional non-adherence due to forgetfulness, not intentional non-adherence. We have opted for this intervention since the majority of Slovak patients report forgetting as the major cause of non-adherence [98]. Moreover, pharmacies are highly accessible in Slovakia and at least one antihypertensive drug within each class is fully reimbursed by the public health insurance. Access to antihypertensive medicines is therefore not an obstacle for patients’ adherence in Slovakia.

Additionally, we collect patients’ health and medication information as patient-reported outcomes. Since pharmacists in Slovakia do not have access to patients’ health or medication history, patients are their only source of this information also in everyday practice. Thus, on the one hand, our pragmatic trial design reflects everyday practice; on the other hand, it also presents a limitation relying on the memory and truthfulness of the patients.

Another limitation of the study is that pill count assessment used alone, as in our secondary analysis, is often not valid. Patients may tend to remove their pills before the visit to please the healthcare provider who performs the pill count. Nevertheless, we have decided to include this outcome in our secondary analysis based on the following rationale. First, pill count as a research method is not frequently used in Slovakia and we will not explicitly inform the participants that their pills will be counted. Hence, participants will not be alerted to this fact. Second, we assume that patients’ primarily want to please their physician, who prescribed them the medicines and not necessarily the pharmacist, viewed more as a trusted counselor, in front of whom there is no need to hide information on medication intake.

The three-month intervention period is relatively short to ensure long-lasting clinical improvement and the potential benefits may wear off with time. However, once the initial clinical benefit, feasibility, patient acceptance, and cost-effectiveness are proven in this trial, we plan to conduct follow-up research to address these limitations.

A limitation to the pragmatic trial design is also that the external unblinded trial pharmacist is sending the SMS reminders centrally. We have accepted this limitation to ensure the blinding of the trial pharmacists as assessors of patients’ adherence, which is the primary objective of the current trial.

The SPPA trial will be the first registered pharmaceutical clinical trial in Slovakia. Understanding the need of evidence-based pharmaceutical care (EBPC) [57, 104], we aim at improving the quality of pharmaceutical care through EBPC also in the currently presented trial. SPPA trial addresses the main healthcare challenges not only in the Slovak Republic: high prevalence of hypertension and other secondary cardiovascular diseases, overconsumption of cardiovascular drugs, alarmingly low AR to long-term medication treatment, and high rates of uncontrolled blood pressure. In countries, whose healthcare systems lack financial resources and healthcare professionals, similar to the Slovak Republic, a simple mHealth intervention in form of SMS reminders of medication intake provided by pharmacists may be an efficient way to address all of these problems.

The SPPA trial will provide essential information to design future studies assessing mHealth technologies through EBPC. Therein, we will concentrate on clinically important outcomes, provided that the SPPA trial shows promising results in improving systolic blood pressure. The focus on objective clinical outcome will also enable directly the pharmacists to send the SMS reminders to patients without the need to remain blinded. Moreover, we will adjust the intervention to better reflect patients’ needs using the collected patient reported outcomes in the presented trial. Detailed publicly available reporting of the SPPA trial will serve as a source of valuable information on methodology, design, and conduct of pragmatic clinical trials in community pharmacies.

## Trial status

This study is currently recruiting participants.

## Additional files

Additional file 1: SPIRIT checklist. Filled out SPIRIT checklist for the SPPA trial protocol. (PDF 114 kb)

Additional file 2: PRECIS-2 table of scores for trial domains and the PRECIS-2 wheel scheme. Filled out PRECIS-2 table of scores and the resulting scheme of PRECIS-2 wheel. (PDF 197 kb)

Additional file 3: SPPA trial forms and materials. All trial forms and materials, including case report forms (in English language). All of them form Appendices to the SPPA trial protocol and were approved by the Ethics Committee together with the SPPA trial protocol. (PDF 5685 kb)

## Abbreviations

AR: Adherence rate
CONSORT: Consolidated Standards of Reporting Trials
EBPC: Evidence-based pharmaceutical care
mERA: Guidelines for reporting of health interventions using mobile phones: mobile health (mHealth) evidence reporting and assessment
mHealth: Mobile health
MMAS-8: Eight-item Morisky Medication Adherence Scale
PRECIS-2: PRagmatic Explanatory Continuum Indicator Summary
QUINT: Qualitative INteraction Trees
SMS: Short messaging service
sPhC: Standard pharmaceutical care
SPIRIT: The Standard Protocol Items: Recommendations for Interventional Trials
WHO: World Health Organization
