# Supplementary material for: The effectiveness of daily SMS reminders in pharmaceutical care of older adults on improving patients’ adherence to antihypertensive medication (SPPA): study protocol for a randomized controlled trial
Source: Trials. 2017 Jul 18;18:334. doi: 10.1186/s13063-017-2063-8 (PMC5516377; doi:10.1186/s13063-017-2063-8)
Supplement: Supplementary file 2 — PRECIS-2 table of scores for trial domains and the PRECIS-2 wheel scheme. Filled out PRECIS-2 table of scores and the resulting scheme of PRECIS-2 wheel. (PDF 197 kb) [file 13063_2017_2063_MOESM2_ESM.pdf]

PRECIS-2 table of scores for trial domains

	Domain	*Score*	*Rationale*
**1**	Eligibility Criteria	5	The assessed intervention is intended for older (≥ 55 years) patients to help them improve their adherence to antihypertensive drugs intake. This group of patients usually has the highest cardiovascular risk and thus could benefit from the strengthening of adherence to antihypertensive medication the most.<br>We are including only patients who take antihypertensive drugs for more than a year since we are focusing on assessing the impact of the intervention on patients who have already accepted their medication therapy (excluding early discontinuation of medication treatment due to non-acceptance of the therapy itself). These patients are also our target group, should the intervention be implemented as a part of the usual pharmaceutical care.<br>The intervention is limited to patients who own a mobile device, have the ability to open and read SMS and have no biological impairment affecting the ability to read the SMS. However, the very nature of the evaluated intervention rules out the use in these groups of patients.<br>Patients who are hospitalised during the trial are excluded from the trial since a medication intake reminder system is redundant while a patient is in the hospital, where nurses provide him/her regularly with his/hers medicines.
**2**	Recruitment Path	5	Recruitment takes place in community pharmacies during a regular patient visit with the purpose of filling their antihypertensive drugs prescription(s). Potential trial participants visit the pharmacy on their behalf, without any special incentive.<br>Moreover, we plan to recruit trial participants from 6 different pharmacies. To maintain applicability and generalizability of the results for the whole Slovak population of older hypertensive patients, selected trial pharmacies will be evenly distributed to cover the whole territory of Slovakia. Additionally, half of the selected community pharmacies will be located in rural, and the other half will be located in the urban region.
**3**	Setting	5	Identical setting to the usual care setting – community pharmacies, where patients regularly collect their antihypertensive medications.
**4**	Organisation of Intervention	3	The trial evaluates the impact of the additional intervention (SMS reminders of medication intake) provided to the patients along with the usual pharmaceutical care. This intervention in not currently being used in the usual care. This difference is an explicit feature of the intervention.<br>However, the intervention could be easily implemented in the usual care. The required financial and time resources are significantly low. Potential necessary training of the pharmacists is brief and the software itself is easy to use and highly self-explanatory. Also, the majority of the patients are already familiar with SMS usage and thus the intervention does not pose any potential problems with adaptation.<br>Even though the intervention is provided in the trial by an additional pharmacist (external unblinded trial pharmacist), this is only to ensure that the investigators remain blinded to intervention allocation thorough the trial.

**5**	Flexibility of experimental intervention - delivery	5	The information provided in the SMS reminder (intervention) mirror those provided as a part of the usual drug dispensation and counselling process as described in the Slovak legislation (Decree No. 129/2012).<br>All pharmacists that have successfully completed their pharmaceutical education (graduated as "Masters of Pharmacy") are well trained and highly competent to provide professional information on medication intake. This health service is a compulsory part of their usual Pharmaceutical Care provision.
**6**	Flexibility of experimental intervention - adherence	-1	This domain is not relevant for the SPPA trial. The assessed intervention of the trial is aimed at increasing patients' adherence to blood pressure-lowering medication intake.
**7**	Follow up	4	The follow-up takes place after 3-months period, which is the usual time for a regular doctor appointment for the long-term hypertension patients (trial population).<br>According to the Slovak legislation, Act No. 362/2012 Z. z., §120, d), the number of prescribed packages of a drug is not allowed to exceed the number of doses required for three months of treatment for patients who are taking the drug on a regular basis. Thus, hypertensive patients who take their blood pressure-lowering medicines on a long-term basis are visiting the pharmacy usually in 3 months cycles.<br>However, to maintain the attrition of participants at a low rate, trial pharmacists will contact all participants via phone call 2-3 days before their pre-scheduled follow-up visit as a reminder. During this call, the follow-up visit can be rescheduled to suit the need of the patient. If the patient is for some reason not able to attend the follow-up visit, the MMAS-8 and satisfaction questionnaire can be completed via phone call.<br>These follow-up-encouraging phone calls support our endeavour to receive feedback on the intervention directly from the patients in the intervention group.
**8**	Outcome	3	Our primary outcome is the proportion of adherent patients. Poor medication adherence is considered the main factor for uncontrolled blood pressure resulting in increased risk of stroke, hospitalisation and premature death. In Slovakia, this is a major health issue. Moreover, WHO concludes that improving patients' adherence to medication may have a greater effect on the health than any other improvement in therapy.<br>Despite the proven association between high adherence to medication intake and good blood pressure control, the primary outcome itself is not a priority for the patients, but it is a condition for almost all other relevant patients outcomes since medicines work only in patients who actually take them.<br>Our primary outcome is therefore of the utmost relevance mainly for the commissionaires of care and policymakers.
**9**	Analysis	5	Intention-to-treat analysis

PRECIS-2 evaluation of the SPPA trial
Available online: https://www.precis-2.org/Trials/Details/251

PRECIS-2 wheel scheme of the SPPA trial

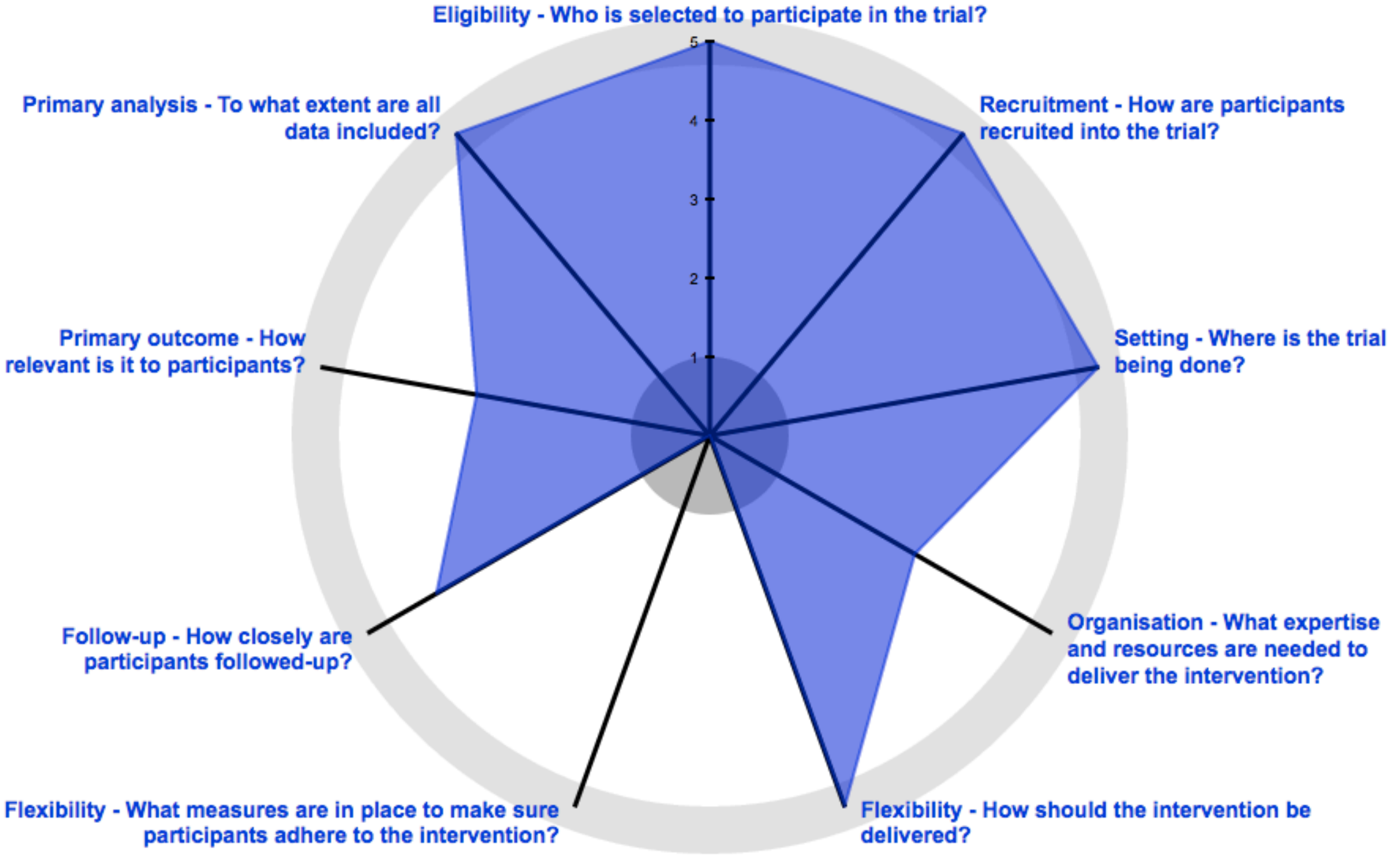

PRECIS-2 evaluation of the SPPA trial
Available online: https://www.precis-2.org/Trials/Details/251
