# Supplementary material for: The effectiveness of daily SMS reminders in pharmaceutical care of older adults on improving patients’ adherence to antihypertensive medication (SPPA): study protocol for a randomized controlled trial
Source: Trials. 2017 Jul 18;18:334. doi: 10.1186/s13063-017-2063-8 (PMC5516377; doi:10.1186/s13063-017-2063-8)
Supplement: Supplementary file 3 — SPPA trial forms and materials. All trial forms and materials, including case report forms (in English language). All of them form Appendices to the SPPA trial protocol and were approved by the Ethics Committee together with the SPPA trial protocol. (PDF 5685 kb) [file 13063_2017_2063_MOESM3_ESM.pdf]

Appendix 1: Case Report Form Visit 1 - PART A

Case Report Form Visit 1 - PART A

Personal and Medication Information

Patient Trial Code:	
Birth Certificate Number:	
Address of Residency:	
Mobile Telephone Number:	
Date of Visit 1:	

Blood pressure-lowering drug(s):

**No.**	**Drug Name** (Brand Name)	**No. of packages dispensed**	**No. of pills in one package**	**Strength** (mg)	**Dosage Regimen** (e.g. 1-0-1)	**Any pills left at home** (YES/NO)	**If YES, how many** (No. /PDK*)	**Further information** (Daytime, before/with/after meal, etc.)
**1**								
**2**								
**3**								
**4**								
**5**								
**6**								
**7**								
**8**								
**9**								
**10**								

Date of scheduled Visit 2:	______ . ______. 2017 (*at least 3 months after Visit 1*)	***Preferred time for daily SMS reminders:***	________:________ (*please, use 24 hour format*)

**PDK = Patient doesn't know → ! A phone call with the patient needs to be scheduled to find out the number of pills left at home !*

Case Report Form *Visit 1 - PART B*<br>**Blood pressure measurement**	
Patient Trial Code:	
Date of Visit 1:	

**1st BP measurement – LEFT ARM**	
systolic BP [mmHg]	
diastolic BP [mmHg]	
**1st BP measurement - RIGHT ARM**	
systolic BP [mmHg]	
diastolic BP [mmHg]	
**2nd BP measurement on the arm with higher BP**	
systolic BP [mmHg]	
diastolic BP [mmHg]	
If there is a disparity in the 1st and 2nd measurement, perform a 3rd measurement on this arm:<br>**3rd BP measurement on the arm with higher BP**	
systolic BP [mmHg]	
diastolic BP [mmHg]	

**Final BP (average of measurements from the arm with higher BP)**	
systolic BP [mmHg]	
diastolic BP [mmHg]	

Appendix 3: Case Report Form Visit 1 - PART C

Case Report Form *Visit 1 - PART C*<br>**Medication Adherence Measurement (MMAS-8)**	
Patient Trial Code:	
Date of Visit 1:	

You indicated that you are taking medication(s) for your high blood pressure.

Individuals have identified several issues regarding their medication-taking behavior, and we are interested in your experiences.

There is no right or wrong answer.

Please answer each question based on your personal experience with your blood pressure-lowering medication(s)

1. Do you sometimes forget to take your blood pressure-lowering medication(s)?

YES NO

2. People sometimes miss taking their medication(s) for reasons other than forgetting. Thinking over the past two weeks, were there any days when you did not take your blood pressure-lowering medication(s)?

YES NO

3. Have you ever cut back or stopped taking your medication(s) without telling your doctor, because you felt worse when you took it?

YES NO

4. When you travel or leave home, do you sometimes forget to bring along your blood pressure-lowering medication(s)?

YES NO

5. Did you take your blood pressure-lowering medication(s) yesterday?

YES NO

6. When you feel like your blood pressure is under control, do you sometimes stop taking your medication(s)?

YES NO

7. Taking medication(s) every day is a real inconvenience for some people. Do you ever feel hassled about sticking to your blood pressure-lowering treatment plan?

YES NO

8. How often do you have difficulty remembering to take all your medication(s)?

1. Never/Rarely
2. Once in a while
3. Sometimes
4. Usually
5. All the time

Appendix 4: Case Report Form Visit 1 - PART D

Case Report Form *Visit 1 - PART D*

Patient information form

Patient Trial Code:	
Date of Visit 1:	

Please mark with a cross the applicable answer *(Your answer will remain anonymous)*

example : [x]

Should you have any questions, please do not hesitate to ask you trial pharmacist, he/she will gladly assist you.

1) Sex

Please, mark whether you are female or male.

female	
male	

2) Education

Please, mark the highest level of education, you have achieved.

primary school	
high school (grammar school)	
university undergraduate (BSc)	
university undergraduate (MSc)	
university postgraduate (PhD)	
I don't wish to disclose this information	

3) Monthly Income

Please, mark in which of the ranges your monthly income belongs.

< 300 EUR	
300-600 EUR	
601-800 EUR	
801-1000 EUR	
> 1000 EUR	
I don't wish to disclose this information	

4) Marital Status

Please, mark the option best best describing your current marital status.

single	
married or in a long-term partner relationship	
divorced	
widowed	
I don't wish to disclose this information	

5) Living Arrangement *Please, mark the option best describing your current living arrangement.*	
living with my partner/ husband (wife)	☐
living with my family or friends	☐
living in a nursing home	☐
living alone	☐
I don't wish to disclose this information	☐
6) Hypertension Duration *Please, mark how many years before now your doctor prescribed you blood pressure-lowering medicines for the first time.*	
< 5 years	☐
5-10 years	☐
> 10 years	☐
7) Smoking Status Please, mark the option best describing your current relationship to smoking.	
non-smoker	☐
current smoker	☐
ex-smoker	☐
I don't wish to disclose this information	☐
8) Current number of daily medications Please, mark the number of drugs you are taking per day (please, consider all your current drugs.	
< 2	☐
3-4	☐
5-6	☐
7-8	☐
> 8	☐
9) Concomitant diseases *Has a doctor or nurse ever told you that you have (please, tick all that apply):*	
high cholesterol	☐
diabetes	☐
atherosclerosis	☐
heart failure	☐
arthritis	☐
back pain	☐
asthma	☐
any other disease	☐

please, write down any other disease, your doctor or nurse has told you that you have:

10) Use of pillboxes

Please, mark if you use pillboxes to help you remember to take your medicines in time.

NO	☐
YES	☐

11) Use of other reminder devices

Please, mark if you use any other devices to help you remember to take your medicines in time?
(e.g. alarm-clock, reminders from family members, etc.)

NO	☐
YES	☐

if YES, what kind of reminder system:

12) Purpose of using a mobile phone

Please, mark the most relevant way you use your mobile phone.

mostly personal	☐
mostly professional	☐
personal and professional	☐
I don't wish to disclose this information	☐

13) Receiving SMS

Please, mark the approximate number of SMS you received last year

one or more SMS a day	☐
approximately one SMS a week	☐
approximately one SMS a month	☐
less than one SMS a month	☐
I don't wish to disclose this information	☐

14) Sending SMS

Please, mark the approximate number of SMS you sent last year.

one or more SMS a day	☐
approximately one SMS a week	☐
approximately one SMS a month	☐
less than one SMS a month	☐
I don't wish to disclose this information	☐

Thank you very much for your precious time and your participation in the SPPA clinical trial !

Appendix 5: Case Report Form Visit 2 - PART A

Case Report Form Visit 2 – PART A<br>Pill Count	
Patient Trial Code:	
Date of Visit 2:<br>*(If patient attended Visit 2 in the pharmacy)*	
Date of phone call:<br>*(If pill count performed via phone call)*	

No.	**Drug Name (Brand name)**	**No. of remaining pills in the marked package**	**pill count performed in pharmacy**	**pill count performed via phone call**	**Initials of team member performing pill count/ phone call**	**Further comments**
1.						
2.						
3.						
4.						
5.						
6.						
7.						
8.						
9.						
10.						

*Please, make sure all evaluated drugs (marked packages) of each patient are listed.
In case the patient forgot to bring the package(s) to the pharmacy, try to arrange a follow up phone call with the patient to count the pills during this phone call on the same day. If not possible, for this drug/ these drugs list only the reason why it was not possible to collect in the filed "Comments".

Appendix 6: Case Report Form Visit 2 - PART B

Case Report Form Visit 2 – PART B<br>Blood pressure measurement	
Patient Trial Code:	
Date of Visit 2:<br>**(If patient did not attend Visit 2 at the pharmacy, strike-through the whole CRF Visit 2-PART B form from this patient)**	

**1st BP measurement – LEFT ARM**	
systolic BP [mmHg]	
diastolic BP [mmHg]	
**1st BP measurement - RIGHT ARM**	
systolic BP [mmHg]	
diastolic BP [mmHg]	
**2nd BP measurement on the arm with higher BP**	
systolic BP [mmHg]	
diastolic BP [mmHg]	
If there is a disparity in the 1st and 2nd measurement, perform a 3rd measurement on this arm:<br>**3rd BP measurement on the arm with higher BP**	
systolic BP [mmHg]	
diastolic BP [mmHg]	

**Final BP (average of measurements from the arm with higher BP)**	
systolic BP [mmHg]	
diastolic BP [mmHg]	

Case Report Form *Visit 2 - PART C*<br>Medication Adherence Measurement (MMAS-8)	
Patient Trial Code:	
Date of Visit 2:<br>*(If patient attended Visit 2 in the pharmacy*	
Date of phone call:<br>*(If MMAS-8 was completed via phone call)*	

You indicated that you are taking medication(s) for your high blood pressure
Individuals have identified several issues regarding their medication-taking behavior, and we are interested in your experiences.

There is no right or wrong answer.

Please answer each question based on your personal experience with your blood pressure-lowering medication(s)

1. Do you sometimes forget to take your blood pressure-lowering medication(s)?

YES NO

2. People sometimes miss taking their medication(s) for reasons other than forgetting. Thinking over the past two weeks, were there any days when you did not take your blood pressure-lowering medication(s)?

YES NO

3. Have you ever cut back or stopped taking your medication(s) without telling your doctor, because you felt worse when you took it?

YES NO

4. When you travel or leave home, do you sometimes forget to bring along your blood pressure-lowering medication(s)?

YES NO

5. Did you take your blood pressure-lowering medication(s) yesterday?

YES NO

6. When you feel like your blood pressure is under control, do you sometimes stop taking your medication(s)?

YES NO

7. Taking medication(s) every day is a real inconvenience for some people. Do you ever feel hassled about sticking to your blood pressure-lowering treatment plan?

YES NO

8. How often do you have difficulty remembering to take all your medication(s)?

1. Never/Rarely
2. Once in a while
3. Sometimes
4. Usually
5. All the time

Satisfaction Questionnaire
SPPA trial

We would like to express our gratitude for your participation in our clinical trial!

This questionnaire addresses your experience with the additional pharmaceutical care service of SMS reminders helping you to take your blood pressure-lowering medicines on time. Your answers will guide us in improving the service for you and other patients in the future.

Please, circle the answer that most appropriately describes your opinion

1. Do you feel SMS reminders helped you to remember to take your blood pressure-lowering drugs?

1	2	3	4	5	0
Fully agreed	Rather agreed	Neither agreed nor disagreed	Rather disagreed	Completely disagreed	I don't know

2. Do you feel SMS reminders have disrupted your medication intake?

1	2	3	4	5	0
Fully agreed	Rather agreed	Neither agreed nor disagreed	Rather disagreed	Completely disagreed	I don't know

3. Was the wording of the SMS reminders understandable?

1	2	3	4	5	0
Fully agreed	Rather agreed	Neither agreed nor disagreed	Rather disagreed	Completely disagreed	I don't know

4. Would you suggest the SMS reminders od drug intake to your friends or family members?

1	2	3	4	5	0
Fully agreed	Rather agreed	Neither agreed nor disagreed	Rather disagreed	Completely disagreed	I don't know

5. Was the frequency of the SMS reminders right?

1	2	3	4	5	0
Fully agreed	Rather agreed	Neither agreed nor disagreed	Rather disagreed	Completely disagreed	I don't know

6. Would you like to continue receiving SMS reminders of your blood pressure-lowering medication?

1	2	3	4	5	0
Fully agreed	Rather agreed	Neither agreed nor disagreed	Rather disagreed	Completely disagreed	I don't know

7. **In general, are you satisfied with the additional pharmaceutical service of daily SMS reminders of your blood pressure-lowering medication?**

**1**	**2**	**3**	**4**	**5**	**0**
Fully agreed	Rather agreed	Neither agreed nor disagreed	Rather disagreed	Completely disagreed	I don't know

8. **Would you like to regularly receive also other information related to your health in from of short informational SMS from your pharmacist (e.g. helpful advice about diet, wellbeing, exercise, medication etc.)?**

**1**	**2**	**3**	**4**	**5**	**0**
Fully agreed	Rather agreed	Neither agreed nor disagreed	Rather disagreed	Completely disagreed	I don't know

9. **If you would like to continue receiving SMS reminders of your blood pressure-lowering medication, would you like to receive them:**

**1**	**2**	**3**	**4**	**0**
Once a day	Once a week	Once a month	Before each medication dose	I don't know

Below, you can leave your observations, comments and experiences with the SMS reminders of blood pressure-lowering drug intake:

Thank you very much for your precious time!

Your SPPA trial Research Team

Appendix 9: Sample Randomization and Allocation List

Randomization and Allocation List
SPPA Trial

Trial Centre (Pharmacy):
Address:
Principal Investigator (Pharmacist):

Group	*Female*	*Male*
Intervention group	1659	1203
	1546	1837
	1765	1199
	1970	1954
	1832	1922
	1011	1286
	1350	1577
	1130	1620
	1106	1788
	1604	1250
	1602	1928
	1929	1420
	1620	1471
	1186	1834
	1585	1310
Control group	1281	1529
	1735	1896
	1795	1782
	1571	1383
	1380	1893
	1361	1727
	1387	1469
	1487	1534
	1357	1809
	1211	1034
	1895	1143
	1902	1644
	1594	1209
	1612	1528
	1607	1273

Appendix 10: Sample Randomization envelopes

RANDOMIZATION ENVELOPE
SPPA TRIAL
MEN

PATIENT TRIAL CODE: 1203

Participant's Name: ______________________

Mobile Phone Number: ____________________

RANDOMIZATION ENVELOPE
SPPA TRIAL
WOMEN

PATIENT TRIAL CODE: 1659

Participant's Name: ______________________

Mobile Phone Number: ____________________

Appendix 11: Screening and Enrollment Log

Screening & Enrollment Log
SPPA Trial

Trial Centre (Pharmacy): *Pharmacy Name & Address [Code: XX]*

Principal Investigator (PI): ____________________

Patient Number	***Enrolled (YES or NO)***	***Patient Trial Code (CODE or N/A)***	***Reason for no enrolling (REASON or N/A)****	***Date of ICF signature***	***Initials of investigator***
**XX-001**					
**XX-002**					
**XX-003**					
**XX-004**					
**XX-005**					
**XX-006**					
**XX-007**					
**XX-008**					
**XX-009**					
**XX-010**					

** as reason can be also stated "patient refused to provide a reason"*

Adverse Events Report Form

Sites

SPPA Trial

Trial Centre (Pharmacy): *Pharmacy Name & Address [Code: XX]*

Principal Investigator (PI): ____________________

Contact No.	Date of Contact	Patient Trial Code	Patient reported signal of an adverse event associated with an antihypertensive drug	Study member initials
1.				
2.				
3.				
4.				
5.				

Adverse Events Report Form

Project Leader

SPPA Trial

Contact No.	Date of Contact	Patient Trial Code	Patient reported signal of an adverse event associated with an antihypertensive drug
1.			
2.			
3.			
4.			
5.			

Discontinuation Log

Sites

SPPA Trial

Trial Centre (Pharmacy): *Pharmacy Name and address [Code: XX]*

Principal Investigator (PI): ____

No.	Patient Trial Code	Date of Discontinuation	Reason for Discontinuation in the trial (ONLY if the patient wants to provide the reason)
1.			
2.			
3.			
4.			
5.			

After each discontinuation, inform the Project Leader immediately (NECESSARILY on the date of discontinuation)!

Appendix 15: Discontinuation Log - Project Leader

Discontinuation Log
Project Leader
SPPA Trial

No.	Patient Trial Code	Date of Discontinuation	Reason for Discontinuation in the trial (ONLY if the patient wants to provide the reason)
1.			
2.			
3.			
4.			
5.			

Contact Report Form

Trial Pharmacists - Participants

SPPA Trial

Patient Trial Code: ________________________________

**1st phone call contact (only for patients with spare pills of antihypertensives at home)**		
DATE:* _____ . _____ . *2017		
**blood pressure lowering drug**		**number of pills left at home**
**#1**		
**#2**		
**#3**		
**#4**		
**#5**		
**#6**		
**#7**		
**#8**		
**#9**		
**#10**		

2nd phone call contact (all patients)		
DATE:* _____ . _____ . *2017		
Planned date of Visit 2:	__ __ . __ __ . 2017	
Will the patient attend Visit 2 as planned?	YES	NO
If NO: Date of re-scheduled Visit 2:	__ __ . __ __ . 2017 (if not applicable strike through)	
Patient reminded to bring the marked drug packages:	YES	NO

If the patient is not at all able to attend Visit 2						
**Reason (ONLY if the patient is willing to provide a reason)**	**pill count performed during phone call**		**MMAS-8 questionnaire filed out during phone call**		**Satisfaction questionnaire (if applicable) filed out during phone call**	
	YES	NO	YES	NO	YES	NO

Appendix 17: Contact Report Form - Participants - Sites

Contact Report Form

Participants - Sites

SPPA Trial

Trial Centre (Pharmacy): *Pharmacy Name & Address [Code: XX]*

Principal Investigator (PI): ______________________

Contact No.	Date of Contact	Patient Trial Code	Issue description	Resolution description	Duration (from contact initiation to resolution)
1.					
2.					
3.					
4.					
5.					

Appendix 18: Contact Report Form - Participants - Project Leader

Contact Report Form

Participants – Project Leader

SPPA Trial

Contact No.	Date of Contact	Patient Trial Code	Issue description	Resolution description	Duration (from contact initiation to resolution)
1.					
2.					
3.					
4.					
5.					

Appendix 19: Authorization List

Authorization List
SPPA Trial

Trial Centre (Pharmacy): *Pharmacy Name & Address [Code: XX]*

Principal Investigator (PI): ____________________

Name	*Trial Function*	*Signature*	*Initials*	*Authorized Procedures (No.)*	*Start Date*	*PI Signature/ Initials*	*End Date*	*PI Signature/ Initials*

Legend (No.):

Training Log
SPPA Trial

Trial Centre (Pharmacy): ______________________

Principal Investigator (PI): ______________________

Date of Training: ______________________

**Training Topic(s):**	
**Training Details:**	

Trainee(s):

Name	Signature

Confirmation by Trainer:

______________________	______________	______________________
Name	***Date***	***Signature***

Appendix 21: SPPA Scheme for Blood Pressure Measurement

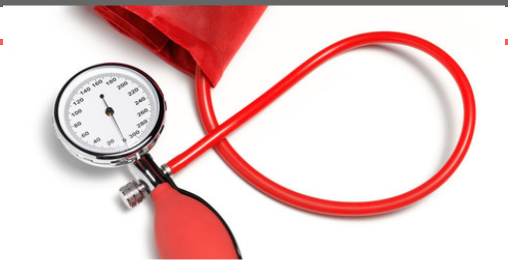

SPPA Scheme
for Blood Pressure Measurement (BPM)

Before BPM

1. **Let the patient rest in a sitting position for 3-5 minutes.**
   (the patient should have uncrossed legs and be seated comfortably in a chair with his/her back and arm supported)
2. **Choose the appropriate cuff that fits patient's arm properly.**
   (The cuff bladder length should cover at least 80% of patient's arm circumference)
3. **Position the cuff at the heart level of the patient**
   (approximately 2-3 cm above the elbow *(antecubital fossa)* with the marker on the cuff in the middle of patient's inner arm)

BPM

Neither the pharmacist nor the patient should talk during the BP measurement

1. **Measure BP on both arms once to detect differences[1].**
2. **Wait 1-2 minutes and repeat the BP measurement on the arm with the higher BP**
3. *(Perform an additional measurement if there is a disparity between the first and the second measurement)*
4. **Note the blood pressure values in the Case Report Form**

[1] *approximately 20% of individuals may have a difference >10 mm Hg*

Adopted according:

MANCIA, G., R. FAGARD, K. NARKIEWICZ, J. REDÁN, et al. 2013 Practice guidelines for the management of arterial hypertension of the European Society of Hypertension (ESH) and the European Society of Cardiology (ESC): ESH/ESC Task Force for the Management of Arterial Hypertension. J Hypertens, 2013, 31(10), 1925-1938.

PICKERING, T. G., J. E. HALL, L. J. APPEL, B. E. FALKNER, et al. Recommendations for blood pressure measurement in humans and experimental animals: part 1: blood pressure measurement in humans: a statement for professionals from the Subcommittee of Professional and Public Education of the American Heart Association Council on High Blood Pressure Research. Circulation, Feb 8 2005, 111(5), 697-716.

Appendix 22: SPPA Trial Patient Card

Front side:

Patient Card
SPPA trial

This patient is participating in a clinical trial investigating patients' adherence to blood pressure-lowering drugs.
The trial does not involve any investigation products or investigational drugs.

Trial Code:
Trial Sponsor: Faculty of Pharmacy, Comenius University in Bratislava
Trial Centre:

Trial duration: ______________________________

Date of the scheduled follow-up visit:______________________

Patient Trial Code: ______________

Back side:

Please, feel welcome to contact **PharmDr. Zuzana Haramiová**, who is the Project Leader of the clinical trial, in case you have any questions regarding the clinical trial.

Please, also contact her in the following cases:
- you have lost your mobile telephone
- you have changed your mobile telephone number
- you have been hospitalised
- your doctor has changed your blood pressure-lowering medication
- you won't be able to participate in the clinical trial
- you no longer wish to participate in the clinical trial and would like to withdraw your consent

Project Leader: PharmDr. Zuzana Haramiová
Telephone Number:

You can call **anytime you feel need for it.**
The phone call will be of **no cost to you.**

Appendix 23: SPPA Trial Medication Label

SPPA trial

Trial Centre (Pharmacy), Address of the Trial Centre (Pharmacy)
